# Role of Non-Coding RNAs in the Development of Targeted Therapy and Immunotherapy Approaches for Chronic Lymphocytic Leukemia

**DOI:** 10.3390/jcm9020593

**Published:** 2020-02-21

**Authors:** Felice Pepe, Veronica Balatti

**Affiliations:** 1 Department of Cancer Biology and Medical Genetics, The Ohio State University, Columbus, OH 43210, USA; Felice.Pepe@osumc.edu; 2 The Ohio State University Comprehensive Cancer Center, The Ohio State University, Columbus, OH 43210, USA

**Keywords:** CLL, *mir-15/16*, venetoclax, targeted therapy, immunotherapy

## Abstract

In the past decade, novel targeted therapy approaches, such as BTK inhibitors and Bcl2 blockers, and innovative treatments that regulate the immune response against cancer cells, such as monoclonal antibodies, CAR-T cell therapy, and immunomodulatory molecules, have been established to provide support for the treatment of patients. However, drug resistance development and relapse are still major challenges in CLL treatment. Several studies revealed that non-coding RNAs have a main role in the development and progression of CLL. Specifically, microRNAs (miRs) and tRNA-derived small-RNAs (tsRNAs) were shown to be outstanding biomarkers that can be used to diagnose and monitor the disease and to possibly anticipate drug resistance and relapse, thus supporting physicians in the selection of treatment regimens tailored to the patient needs. In this review, we will summarize the most recent discoveries in the field of targeted therapy and immunotherapy for CLL and discuss the role of ncRNAs in the development of novel drugs and combination regimens for CLL patients.

## 1. Chronic Lymphocytic Leukemia

Chronic lymphocytic leukemia (CLL) is the most common adult leukemia in western countries, typically diagnosed in the elderly, with a higher prevalence in males and individuals with a family history of CLL [1]. CLL occurs in two forms, indolent or aggressive, and is characterized by the clonal proliferation of CD-5-positive B-lymphocytes that accumulate in the bloodstream, bone marrow, lymph nodes, and spleen [2]. Most patients with an indolent disease survive for several years without treatment, showing mild symptoms. Aggressive CLL, instead, is lethal if not treated and the prognosis is often poor [3]. Two staging methods for CLL are used: Rai [4] and Binet [5] systems. The Rai classification is based on parameters, such as lymphocytosis, enlarged lymph nodes, splenomegaly, hepatomegaly, anemia, and thrombocytopenia. The Binet staging is based on the presence of anemia or thrombocytopenia and the number of areas involved, defined by the presence of enlarged lymph nodes or organomegaly. Both systems are used to select patients for treatment and clinical trials and to evaluate the progression of the disease [6]. CLL diagnosis is also based on the lymphocytes’ immunophenotype and CLL cells typically express CD5, CD19, and CD23 antigens. Other factors involved in CLL diagnosis and prognosis are the expression of CD38, zeta-chain-associated protein kinase 70 (*ZAP-70*), and mutational status of the immunoglobulin heavy chain variable region genes (*IgVH*) [6]. Both indolent and aggressive forms show the clonal expansion of CD5-positive B-cells [7]. However, aggressive CLLs show high *ZAP-70* expression and unmutated *IgVH* while indolent CLLs show low *ZAP-70* and mutated *IgVH*. Genomic aberrations are present in more than 80% of CLL cases [8]. The most common chromosomal abnormalities detectable by cytogenetic include deletion at 13q14.3 (~55%), deletion at 11q22 (~25%), trisomy of chromosome 12 (~10%–20%), and deletion of chromosome 17p13 (~5%–8%) [9,10,11]. The identification of the minimal deleted region (MDR) on the short arm of chromosome 13 in CLL patients was the first indication that the leukemic transformation is initiated by a defect in the apoptotic mechanism driven by the loss of a non-coding RNA gene cluster [12]. Indeed, the 13q14.3 MDR contains the gene cluster encoding for *miR-15a/miR-16-1* [13], which regulates the B-cell lymphoma 2 gene (*BCL2*), a crucial player for apoptosis initiation [14]. Loss of *miR-15a/miR-16-1* prompts overexpression of *BCL2*, which inhibits apoptosis, leading to CLL cell accumulation [15]. Furthermore, *miR-15a/miR-16-1* also target another gene involved in cell growth, the tyrosine-protein kinase transmembrane receptor 1 (*ROR1*), a surface antigen that binds Wnt5a, activating the non-canonical pathway to induce cell proliferation [16]. In this scenario, additional genomic aberration can arise, and indeed, most patients with 11q22 or 17p13 deletions also show the 13q14 deletion. The MDR of chromosome 11 includes the ataxia-telangiectasia mutated gene (*ATM*), involved in the response to DNA double-strand breaks [17]. Similarly, in 17p13-deleted CLL cells, another key regulator of the cell cycle and response to DNA damage is lost: *TP53*. The deletions affecting chromosomes 11 and 17 are mostly associated with aggressive disease while the presence of the 13q deletion as a sole abnormality is associated with an indolent presentation [10]. In addition, genetic mutations of regulatory genes, such as *TP53* [18], *NOTCH1* [19], and *ATM* [20], and aberrant expression of other microRNAs are associated with CLL pathogenesis, drug resistance development, and relapse [21].

## 2. Non-coding RNAs in CLL

In humans, only 1.5% of the genome encodes for proteins [22]. However, ~90% of the total genome is actively transcribed [23]. Most transcripts are non-coding RNAs (ncRNAs) and have a role in various biological processes [24,25]. Infrastructural ncRNAs, such as tRNA and rRNA, are components of the translational machinery [26], while regulatory ncRNAs modulate gene expression [24]. NcRNAs are divided into two categories based on their length: Long ncRNAs (lncRNAs > than 200bp), and small non-coding RNAs (sncRNAs < than 200 bp), including microRNAs (miRNAs), Piwi-interacting RNAs (piRNAs) circRNA, and tRNA fragments. SncRNAs expression is tissue-specific, and these molecules are important regulators of gene expression both at a pre- and post-transcriptional level [27,28] and the first evidence that sncRNAs have a key role in cancer was described in 2002 [13]. This study showed that the microRNA cluster *miR-15a/miR-16-1* is located in the MDR of 13q14.3 observed in most CLL cases. Since then, miRNAs have been widely studied in cancer. MiRNAs are 18–28 ribonucleotide ncRNAs that regulate gene expression by promoting mRNA degradation or by inhibiting mRNA translation [29]. In 2005, a signature of microRNAs was associated with CLL prognosis [30], and in the same year, *miR-15a/miR-16-1* loss was identified as a driver event in CLL onset [31]. In that study, Cimmino et al. demonstrated that *miR-15a/miR-16-1* targets *BCL2*, a key gene involved in the regulation of apoptosis [15,31]. Several years later, it was revealed that *miR-15a/miR-16-1* also targets *ROR1*, the receptor for Wnt5a that initiates the Wnt non-canonical growth signaling pathway [32]. Mutations and microdeletion leading to loss of function of *miR-15a/miR-16-1* were also found not only in CLL (~90%) but in other cancers as well [33,34]. Following those discoveries, several studies identified other dysregulated miRNAs in CLL [21]. In 2006, *miR-29* was found downregulated in aggressive CLL when compared to indolent CLL [35]. However, in 2010, *miR-29a* and *miR-29b* were found overexpressed in indolent CLL when compared to normal CD19^+^ B-cells [36]. Interestingly, *miR-29b* targets the oncogene T-cell leukemia/lymphoma 1 (*TCL1*) whose overexpression leads to aggressive CLL [35]. Thus, dysregulation of *miR-29* alone is not sufficient to develop an aggressive disease, but since *miR-29* targets *TCL1* [37], its downregulation in aggressive CLL may contribute to the overexpression of *TCL1*, leading to the development of an aggressive disease [36]. In 2007, Auer et al. indicated that the *miR-34b/c* cluster is deleted in 11q-CLL [38], and in 2008, Lehmann et al. described an 11q^-^ CLL case where an additional microdeletion was affecting the remaining allele of *miR-34b/c* [39]. Later, the same group showed that the remaining allele of *miR-34b/c* is often epigenetically silenced in most 11q^-^ CLL cases [40]. Interestingly, *miR-34a*, residing on chromosome 1, is dysregulated in many cancers [41], and frequently downregulated in fludarabine-refractory CLLs [42]. Thus, in CLL, *miR-34b/c* are mostly lost or epigenetically silenced. Furthermore, *miR-34a* and *miR-34b/c* are involved with *miR-15a/miR-16-1* and *TP53* in a feedback loop that explains the indolent presentation of 13q^-^ when compared to 11q^-^ aggressive CLL [43]. Indeed, *TP53* positively regulates both *miR-34a* and *miR-34b/c*, and the loss of *miR-34* expression is associated with resistance against apoptosis induced by *TP53*-activating agents [44]. Interestingly, *miR-34a* targets *AXL* [45], encoding for a receptor tyrosine involved in cell proliferation and survival [46]. Activation of *TP53*, in CLL cells with functional *TP53*, inhibits *AXL* expression by activating *miR-34a* transcription. In contrast, CLL B-cells with a non-functional *TP53* show high levels of *AXL* because *miR-34a* is not transactivated. As a result, CLL B-cells with 17p13 deletion express higher levels of *AXL* when compared to those with no 17p deletion. Thus, *AXL* is an attractive therapeutic target in CLL patients with 17p13 deletion and has the potential to be an effective therapeutic target in CLL B-cells regardless of the *TP53* and *miR34* status [45]. Remarkably, the downregulation of *miR-34a* was recently also associated with Richter’s syndrome [47]. Altogether, these data suggest that a compound mimicking *miR-34a* or *miR-34b/c* could be studied for CLL treatment. In 2009, Calin et al. found that at least 50% of microRNA genes are mutated and located near fragile sites, deleted regions, or common breakpoints, again indicating that miRNAs may be valuable tools for diagnostic and treatment purposes [48]. In the same year, a karyotype-specific microRNA signature was described in CLL patients [49], and the mechanism for the activation of the vascular endothelial growth factor (*VEGF*)-based autocrine pathway in CLL B-cells was elucidated, involving *miR-92-1* (also known as *miR-92a-3p*), which is overexpressed in CLL B-cells. In this report, *miR-92-1* was found to target the Von Hippel–Lindau transcript (pVHL) that, in turn, is responsible for *HIF-1α* degradation [50]. The accumulation of *HIF-1α* facilitates the formation of an active complex at the *VEGF* promoter to induce its expression and secretion [50]. CLL B-cells express *VEGF* receptors and respond to *VEGF* stimuli by upregulating the myeloid cell leukemia 1 gene (*MCL1*) and the X-linked inhibitor of apoptosis protein *XIAP*. Thus, *miR-92-1* overexpression enhances the *VEGF* autocrine pathway for cell survivorship, suggesting that *VEGF* inhibition may be a promising new therapeutic approach in CLL [51,52]. In 2011, *miR-181b* expression was studied in CLL patients during disease progression. This report showed that in sequential samples taken from CLL patients with a progressive disease, the expression of *miR-181b* decreases from the indolent to the aggressive stage, whereas sequential samples taken from patients with a stable indolent disease show a steady expression of *miR-181b*, suggesting a diagnostic value for this microRNA [53]. Later on, *miR-181b* was shown to target *BCL2* and *TCL1* genes [35,54,55], and high *miR-181a/b* expression was associated with better response to chemotherapy [56]. In 2012, Tili et al. described the downregulation of *miR-125b* in CLL, mapping on to chromosome 11q24 near the epicenter of the deleted region in 11q^-^ CLLs [57]. However, a subsequent study showed that the *miR-125* family is implicated in a wide variety of cancers as either repressors or promoters and that upregulation of *miR-125b* in other hematological malignancies is associated to disease progression [58]. In addition, *miR-125a* targets *TP53* [59], and very recently, *miR-125a* upregulation was also found to be associated to Richter’s syndrome along with *miR-34a* downregulation [47]. These conflicting data indicate that *miR-125a* and *miR-125b* may have a similar role in CLL development as *miR-29* and further studies are needed to evaluate the role of these microRNAs in CLL. In 2013, *miR-155* overexpression was found associated with CLL [60]. This report indicates that *miR-155* levels gradually increase as normal B-cells progress to monoclonal B-cell lymphocytosis and to CLL, and pre-treatment plasma levels of *miR-155* were found to be lower in patients who experienced a complete response when compared to those that did not. In 2014, *miR-155* overexpression was associated with aggressive CLL [61]. The expression of *miR-155* is positively regulated by environmental factors, such as BAFF signaling, and its activation can enhance the B-cell receptor (BCR) signaling, promoting proliferation in cancer cells [61]. Additionally, *miR-155* was also associated to aneuploidy and early cancer transformation, indicating that its overexpression is an early event in disease onset [62] and that this microRNA could represent a valuable target for therapy. In the same year, *miR-150* was found to be involved in the BCR signaling regulation in CLL by regulating *GAB1* and *FOXP1* [63].

Lastly, the dysregulation of tRNA-derived small non-coding RNAs (tsRNAs) was very recently described in CLL and other cancers. In 2015, *miR-3676* was found to be downregulated in all types of CLL and co-deleted with *TP53* in 17p^-^ CLL [64]. Additionally, *TCL1* is a confirmed target of *miR-3676*, suggesting that this molecule has a key role in the development of aggressive CLL. Later on, it was revealed that *miR-3676* is a tsRNA, generated during the tRNA maturation process [65]. Interestingly, tsRNAs can interact with both Ago and Piwi proteins, affecting the regulation of gene expression at both pre- and post-transcriptional levels [65,66]. In 2017, the expression of tsRNAs in cancers was studied and found to be dysregulated in several types of malignancies [65]. In 2019, tsRNAs and other types of tRNA fragment were found dysregulated in CLLs, thus possibly representing additional diagnostic tools. More research is warranted to evaluated tsRNAs targets as possible targets for the new therapeutic strategies [67].

The dysregulation of different types of sncRNAs in cancer and their role of in the fine tuning of genes and pathways that can be targeted by specific anti-cancer drugs, is crucial. This discovery opened two new fields of study: the identification of specific sncRNAs signatures in cancer to use as diagnostic tools, and that identification of their targets to evaluate as targets for the development of new therapeutic strategies. Indeed, the discovery that altered expression of sncRNAs is associated with the dysregulation of genes and pathways with a key role in CLL onset, progression, and drug resistance, led to the development of several novel compounds to treat CLL and possibly other malignancies, by targeting cancer-specific molecules involved in apoptosis and cell growth [32]. Some of these compounds are small molecules that target proteins essential for cancer cell survival. Other compounds are immunotherapeutic agents such as monoclonal antibodies against specific cancer markers. Both targeted and immunotherapeutic strategies are nowadays at the forefront of cancer treatment.

## 3. NcRNAs, Tumor Microenvironment, and Extracellular Vesicles in CLL

Extracellular vesicles (EVs) are lipid bi-layered particles naturally released into the extracellular environment by many cells, including cancer cells, and carrying a cargo of several molecules from the parent cell. EVs have several biological functions, including the transfer of functional proteins and ncRNA. EVs can be taken up from recipient cells in several ways: receptor–ligand interactions, fusion with the target cell membrane, or internalization by endocytosis [68]. It has been suggested that cancer cell-derived EVs may stimulate the tumor microenvironment (TME) to support tumor growth and spread, and that microRNAs transferred via EVs may target specific pathways that induce a prometastatic inflammatory response [69]. In turn, tumor-associated macrophages can act as nurse-like cells (NLC) and prevent CLL B-cells from apoptosis [70,71]. Thus, since then, circulating microRNAs in body fluids have been proposed as new biomarkers and possible targets for the development of new therapy delivery systems.

In 2013, Umezu et al. showed that EV miRNAs have a key role in leukemia-endothelial cells’ crosstalk [72]. In 2015, a study was carried out to characterize CLL-derived EVs, revealing that the α-IgM-driven activation of the BCR induces CLL B-cells to release EVs, whereas BCR inactivation via ibrutinib inhibits α-IgM-stimulated EV release. The same study also showed a microRNA profiling of the plasma CLL-EVs, identifying a signature that includes the *miR-29* family, *miR-150*, and *miR-155*, the expression of which increases with BCR activation [73]. In addition, cellular and serum levels of *miR-150* were associated with the opposite clinical prognoses and suggested as a molecular prognostic factor in CLL progression [74].

Several additional major components of the TME were described in the last few years, including myeloid-derived suppressor cells (MDSCs), the activation of which in CLL suppresses T-cell responses. MDSCs are generated in the bone marrow and, in tumor-bearing hosts, migrate to the tumor site to support the establishment of the TME [75]. Bruns et al. showed that *miR-155* delivered by CLL-EV to MDSC for induction can be disrupted by vitamin D [76].

Lastly, it is interesting to notice that even though the miRNA cargo in EVs largely represents the cell of origin, selective enrichment for specific microRNAs can occur, as observed for *miR-202-3p* in CLL [77]. Since a tumor suppressor role of *miR-202-3p* was suggested by a previous study showing that this miR is downregulated in follicular lymphoma [78], it was suggested that the compartmentalization in EVs of *miR-202-3p* in CLL may represent a system to remove a tumor suppressor. Indeed, in CLL, the release of *miR-202-3p* results in a decrease of its anti-tumorigenic effect within malignant cells.

Thus, CLL-EVs may influence the disease behavior by affecting both the donor and the recipient cells, as also described by Paggetti et al. [79]. In this report, it is described how CLL EVs taken up by endothelial cells increases angiogenesis and promotes disease progression by inducing the surrounding stromal cells to acquire features of cancer-associated fibroblasts. For these reasons, EVs are currently under investigation as potential drug delivery systems [80,81].

## 4. Treatment Options for CLL

CLL patients are selected for treatment when showing an aggressive symptomatic disease. Chemotherapy alone is no longer used for CLL treatment. However, chemotherapeutic agents are still administered to CLL patients in combination with immunotherapeutic agents and, less frequently, with targeted therapy. Examples of chemotherapy agents used in CLL are purine analogs, such as fludarabine, pentostatin, or cladribine; alkylating agents, such as cyclophosphamide, bendamustine or chlorambucil; and corticosteroids [82]. Unfortunately, the cytotoxicity of chemotherapeutic agents is not cancer specific, and thus these compounds damage normal cells, inducing severe side effects. Therefore, newer treatment approaches based on drugs targeting cancer-specific molecules, are being evaluated to provide better response and milder side effects. Targeted therapy can be administrated alone or in combination with chemotherapy and the recent approval of a wide array of exceptionally efficient targeted therapeutic agents led to a reduction in the use of chemoimmunotherapy regimens (FDA approved drugs and clinical trials are reported in Table 1).

Targeted therapies are designed to target proteins expressed by cancer cells and are essential for their survival but not for that of normal cells. The most commonly used targeted therapy approaches for CLL treatment are tyrosine kinase inhibitors (such as BTK and PI3K inhibitors) and Bcl2 blockers (such as venetoclax). Immunotherapy is designed to stimulate the immune system to target cancer cells. Immunotherapy options currently available for CLL are mainly represented by monoclonal antibodies (often used in combination with chemotherapy), and the more recently developed adoptive cell therapy, such as chimeric antigen receptor T-cell (CAR T-cell). Studies on checkpoint inhibitors’ and immunomodulators’ efficacy in CLL treatment are also ongoing.

Targeted therapy and immunotherapy can be used alone or in combination with chemotherapy. Although highly effective, chemo-targeted therapy and chemo-immunotherapy have side effects and do not always induce a complete remission (CR) without minimal residual disease. Furthermore, patients can develop drug resistance and relapse [83] as a consequence of the selective pressure provided by the therapeutic agent on initially undetectable subclones [84]. Studies on the intratumoral population evolution are ongoing [85]; however, our understanding of the tumor sub-clonal distributions and response to therapy remains limited and it is still not possible to anticipate which clone will progress [86]. Thus, numerous clinical trials are evaluating targeted-immuno combination therapy to provide more tailored and efficient approaches. In the next sections, we will focus on the most recent targeted and immunotherapeutic strategies for the treatment of CLL.

### 4.1. Targeted Therapy for CLL

The National Cancer Institute defines targeted therapies as treatments designed to interfere with the activity of molecular targets associated specifically with cancer cells. In this section, we will describe the newest targeted agents for the treatment of CLL. Table 2 shows the most recent clinical trials of the therapeutic strategies discussed in this review.

#### 4.1.1. Bruton’s Tyrosine Kinase Inhibitors (BTKis)

The Bruton’s tyrosine kinase (BTK) is a key component of the BCR signaling. BTK expression is upregulated in CLL cells and targeting BTK leads to cytotoxicity, inhibition of proliferation/cell migration, and disruption of cytokine/chemokine signaling [87]. Ibrutinib was the first BTK inhibitor approved by the Food and Drug Administration (FDA) for CLL treatment in 2014 [88,89] and it is used for frontline therapy of newly diagnosed CLL patients who require immediate treatment [90], and for relapsed/refractory CLL [91]. Ibrutinib binds to BTK, blocking the BCR signaling, inducing apoptosis, and preventing CLL cells from responding to microenvironment stimuli of survival [92]. Albeit effective, ibrutinib treatment has unique toxicity that can be explained by off-target effects that inhibit other tyrosine kinases [93]. Thus, second-generation BTK inhibitors were designed to reduce toxicity/off-target effects. Among these, acalabrutinib, approved by FDA as monotherapy for CLL in November 2019, offers a higher selectivity in the inhibition of BTK, less adverse effects, and improved efficacy in 17p-deleted cases [94,95] (NCT02029443). Zanubrutinib, approved by the FDA in November 2019, and tirabrutinib are currently under investigation. Zanubrutinib as a single agent is being assessed in CLL/SLL patients intolerant to prior treatment with ibrutinib (NCT04116437) and in comparison with ibrutinib in patients with relapsed/refractory CLL (NCT03734016). The combination of zanubrutinib with obinutuzumab (an anti-CD20 described in Section 4.2.1) plus venetoclax (NCT03824483) or with bendamustine plus rituximab (an anti-CD20 described in Section 4.2.1) are also being evaluated in patients with previously untreated CLL or SLL (NCT03336333). Tirabrutinib is currently under investigation in four active clinical trials for the treatment of B-cell malignancies, and one of them is specifically designed to evaluate safety and efficacy when used in combination with idelalisib (a PI3K inhibitor described in Section 4.1.2) with or without obinutuzumab (NCT02968563).

Acalabrutinib, zanubrutinib, and tirabrutinib limit off-target toxicity but do not overcome the development of ibrutinib resistance, often arising as a consequence of the selection of BTK-mutant clones. Specifically, mutations affecting the C481 residue impair ibrutinib affinity for BTK [96,97]. Thus, the reversible BTKis vecabrutinib (NCT03037645) and LOXO-305 (NCT03740529) were designed to target both the wild-type BTK and the mutated BTK-C481S forms. In addition, mutations of PLC*γ*2 are also associated with ibrutinib resistance [98]. Interestingly, preclinical studies showed that the non-selective reversible BTKi ARQ-531 (NCT03162536) binds BTK without interacting with the C481 residue and also inhibits kinases involved in the downstream BCR signaling, thus possibly retaining its activity despite mutations within PLC*γ*2 [99].

#### 4.1.2. Phosphoinositide 3-Kinase Inhibitors (PI3Ki)

The phosphoinositide 3-kinase (PI3K) signaling pathway regulates cell proliferation and is often altered in CLLs and other cancers [100]. PI3Ks are implicated in cell signaling (class I, II) and membrane trafficking (class II, III). Class I PI3Ks are the most involved in cancer and four isoforms can be identified (α, β, δ, or γ) [101]. In CLL, activation of PI3K-δ and PI3K-γ is a consequence of BCR stimulation, which leads to inhibition of apoptosis and cell survival [102,103,104]. Idelalisib, used for the treatment of relapsed CLL, targets PI3K-δ, induces apoptosis, and prevents the proliferation of CLL cells. Additionally, idelalisib inhibits cell signaling pathways involved in trafficking and homing of B-cells to the lymph nodes and bone marrow [105]. In combination with bendamustine/rituximab therapy, idelalisib triggered a better response in recurrent CLL (NCT01569295). However, this treatment frequently induced adverse effects [106]. Duvelisib, an inhibitor of PI3K-δ and PI3K-γ approved in 2018 for the treatment of relapsed/refractory CLL and small lymphocytic lymphoma (SLL) [107], was designed to affect the TME. Unfortunately, duvelisib showed serious side effects and it is only administered to patients who did not achieve a satisfactory response after at least two systemic therapies [108]. Finally, umbralisib is a PI3K-δ and casein kinase 1 epsilon (CK1-ε) inhibitor. CK1-ε is a regulator of protein translation and it is crucial for activating non-canonical Wnt5a signaling. Interestingly, CK1-ε modulates T-cell activity, reducing the adverse events observed with previous PI3K inhibitors. Indeed, umbralisib shows improved efficacy in non-Hodgkin lymphoma, with a more favorable safety profile [109], and is currently being evaluated for treatment of CLL (NCT02742090). Additionally, a clinical trial is evaluating the umbralisib–ublituximab (an anti-CD20 monoclonal antibody described in Section 4.2.1) combination in patients with relapsed/refractory CLL or Richter’s syndrome (NCT02535286), indicating that this drug may be used in the most advanced and aggressive stages of the disease.

#### 4.1.3. Bcl2 Blockers

The B cell lymphoma 2 (Bcl2) family proteins are key regulators of the apoptotic processes [110,111]. Bcl2 is an important inhibitor of apoptosis and, in follicular B-cell lymphoma is overexpressed as a consequence of a translocation that place the *BCL2* gene under the control of the immunoglobulin heavy chain locus at the breakpoints of t(14;18) [14]. In CLL, Bcl2 overexpression has been associated to the loss of *miR-15a/miR-16-1*, a driver event in CLL onset [13,31,112,113]. These observations led scientists to design a drug that could target Bcl2 [114]. In 2005 the first inhibitor of Bcl2, ABT-737, was designed. ABT-737 is not bioavailable [115], and thus an orally-available derivative navitoclax was developed [116]. Unfortunately, navitoclax also inhibits Bcl-XL which is essential for platelet survival and clinical trials indicated that this drug causes thrombocytopenia [117]. Thus, a very specific Bcl2 inhibitor was generated: venetoclax. The efficacy of venetoclax in targeting CLL cells is outstanding. Venetoclax induces complete remission (CR) in most CLL patients, even though a single-agent treatment may not eradicate the minimal residual disease [118]. Venetoclax induces such strong apoptotic response in CLL cells that patients need to be closely monitored for tumor lysis syndrome during the initial phases of the therapy [119]. Thus venetoclax treatment starts with a low dose that is increased in time (NCT01328626) [120]. Because of its efficiency in inducing CR, venetoclax was approved by the FDA for the treatment of patients with relapsed/refractory CLL in 2016 [121]. In May 2019, venetoclax was approved for first-line treatment of CLL and SLL in adults, and additional clinical trials of combination therapy with ibrutinib or obinutuzumab, are currently ongoing (NCT02910583) (NCT02242942).

#### 4.1.4. Other Targets: CDK Inhibitors, Mcl1 Inhibitors, Axl Inhibitors

CDKs are involved in transcription regulation, mRNA processing, and cell differentiation, and alteration of cyclin-dependent kinase activity is common in several cancers, including CLL [122]. CDKs bind to cyclins to form cyclin-CDK complexes, which are targeted by CDK-inhibitors. Among the most promising CDK inhibitors, CYC065, targeting CDK 2/5/9, is currently studied for relapsed/refractory CLLs in combination with venetoclax (NCT03739554). TG02, a CDK9 inhibitor, leads to the depletion of survival proteins as Mcl1, resulting in p53-independent apoptosis [123], and a clinical trial for CLL patients resistant to ibrutinib treatment is ongoing (NCT01699152).

Myeloid cell leukemia 1 gene (*MCL1*) is an anti-apoptotic member of the *BCL2* family and its overexpression is often associated with drug resistance and relapse in multiple myeloma patients [124]. Interestingly, overexpression of *MCL1* is also associated with venetoclax resistance [125]. Therefore, new drugs are being developed for CLL treatment to target *MCL1* in combination with the Bcl2 inhibitor. Voruciclib, a CDK9/Mcl1 inhibitor, is under examination for FDA approval as in vitro treatment of voruciclib combined with venetoclax, showed induction of apoptosis of CLL cell models and growth arrest in high risk diffuse large B-cells lymphoma models [126]. AZD5991, a highly selective anti-Mcl1, is currently in clinical development. In vitro studies showed that AZD5991 induces apoptosis [127], and a clinical trial for relapsed/refractory hematological malignancies, including CLL, is ongoing (NCT03218683).

As previously mentioned, Axl inhibition induces apoptosis in CLL B-cells with 17p13 deletion and thus several Axl-inhibitors are being developed [46]. In a recent report, TP-0903 treatment effectively reduced Axl phosphorylation and lowered the expression levels of Mcl-1 in ibrutinib exposed CLL B-cells from patients. TP-0903 was found very effective at inducing apoptosis in CLL B-cells from ibrutinib-exposed patients supporting the use of this drug in relapsed/refractory CLL [128]. Thus, an oral formulation of TP-0903 is currently under evaluation in patients with previously treated CLL (NCT03572634).

### 4.2. Immunotherapy for CLL

A remarkable improvement in the treatment approach to CLL was provided by the development of immunotherapy strategies that modulate the immune system response of patients. In this section, we will describe the most recent advances and effective approaches available for CLL patients.

#### 4.2.1. Monoclonal Antibodies

Monoclonal antibodies designed for CLL treatment are grouped according to the targeted protein. The most relevant are monoclonal antibodies directed against CD20, CD19, CD52, CD37, CD38, CD40, BAFF-R and ROR1. Monoclonal antibodies trigger cancer cell death by either activating the antibody-dependent cell-mediated cytotoxicity (ADCC), the antibody-dependent cellular phagocytosis (ADCP), the complement-dependent cell lysis (CDCL) processes [129], the cytotoxic T-lymphocyte response (CTL) or the Helper T lymphocyte response (HTL) [130]. In ADCC, an effector cell (usually a natural killer cell) lyses the target cell whose membrane-surface antigens are bound by specific antibodies. In ADCP, macrophages destroy the targeted cells by phagocytosis. In CDCL, an antibody-coated target cell recruits and activates components of the complement cascade to form a Membrane Attack Complex (MAC) that prompt to cell lysis. In CTL and HTL T-lymphocytes induce or promote the apoptotic death of the target cells.

Anti-CD20: The most frequently used monoclonal antibodies for CLL treatment are targeting CD20, a marker of normal pre-B and mature B lymphocytes. Rituximab was the first monoclonal antibody approved for the treatment of CLLs [129], introduced in 1998 for frontline therapy [131]. After binding to CD20, rituximab triggers a host cytotoxic immune response against CD20-positive cells. Rituximab is often used in combination with chemotherapy as part of the fludarabine-cyclophosphamide-rituximab (FCR) regimen [132]. Obinutuzumab induce ADCC and caspase-independent apoptosis and a clinical trial for combination treatment with venetoclax showed a significant improvement when compared to the previous combination with chlorambucil [133]. Ofatumumab targets a unique epitope of CD20 [134] and induces a strong response against low-CD20 expressing CLL when compared to rituximab [135] or obinutuzumab [136] triggering CDCL and ADCC [137]. Ofatumumab in combination with chlorambucil is indicated for first-line treatment of CLL patients for whom fludarabine-based therapy is inappropriate [138,139]. Ublituximab is showing promising results in comparison to rituximab [140], and is under evaluation for combination immunotherapy with the anti-PI3K umbralisib to treat relapsed/refractory CLL and Richter’s syndrome (NCT02535286). Lastly, mosunetuzumab is an anti-CD20/anti-CD3 bispecific antibody with two antigen-recognition sites, one for CD20, expressed on the surface of B-cells, and one for the CD3 expressed on the surface of T-cells. Upon administration, mosunetuzumab binds to both T-cells (cytotoxic and helper T-cells) and CD20-expressing tumor B-cells resulting in CTL and HTL response against CD20-expressing tumor B-cells. This drug has entered a clinical trial for evaluation of safety and pharmacokinetic as a single agent and combined with atezolizumab (an anti-PD-L1 described in Section 4.2.3) for treatment of Non-Hodgkin’s Lymphoma (NHL) and CLL (NCT02500407).

Anti-CD19: CD19 is expressed during B-cell development [141]. Inebilizumab [115], MOR208 (formerly known as XmAb5574) [142,143], blinatumomab [144] and MDX-1342 [145] are the main anti-CD19 monoclonal antibodies currently under evaluation for CLL treatment. Inebilizumab [146] induces both CTL response and ADCC [147]. A clinical trial evaluated the combination of inebilizumab with bendamustine or rituximab for the treatment of relapsed/refractory CLL (NCT01466153), showing similar efficacy and acceptable safety profile (PMC3990958). MOR208 induces ADCC and ADCP and was well tolerated in relapsed/refractory CLL (NCT01161511) [142]. MOR208 is also under evaluation in several clinical trials for the treatment of B cell lymphomas, and a combination treatment with lenalidomide (an immunomodulatory agent described in Section 4.2.4) is being assessed in patients with relapsed/refractory CLL, SLL or prolymphocytic leukemia (PLL) (NCT02005289). The most interesting, blinatumomab is an anti-CD19/anti-CD3 bispecific monoclonal antibody recognizing the CD19, expressed on the B-cells, and the CD3 expressed on the T-cells (cytotoxic and helper T-cells). This antibody brings CD19-expressing tumor B-cells and CD-3 expressing T-lymphocytes together, resulting in CTL- and HTL-mediated cell death of CD19-expressing B-lymphocytes [144]. In vitro experiments showed that blinatumomab induces faster CLL death that ibrutinib [148], thus this drug is being evaluated for use in non-Hodgkin lymphomas and CLL (NCT03823365).

Anti-CD52, Anti-CD37, Anti-CD38, and Anti-CD40: CD52 is expressed by B- and T- lymphocytes, granulocytes, monocytes, macrophages, Natural Killer and dendritic cells [149]. Alemtuzumab induces lysis of all CD52^+^ cells, thus targeting many components of the immune system and inducing severe side effects. For this reason, alemtuzumab was withdrawal from the market [11]. CD37 is expressed on B-cells and, to a lesser extent, on T-cells and myeloid cells. In vitro experiments on CLL cells showed that CD37 targeting has a potential immunomodulatory role in CLL [150]. Otlertuzumab induces apoptosis of malignant B-cells and ADCC without damaging T-cells and, in combination with bendamustine, increases the response rate and prolongs the progression-free survival of relapsed/refractory CLL patients (NCT01188681) [151]. CD38 is expressed on B lymphocytes and other hematopoietic cells [152], and its stimulation enhanced BCR-signaling inducing cellular proliferation [153,154]. Recently, daratumumab, an anti-CD38 approved for multiple myeloma, was evaluated for CLL treatment. Preliminary data showed that, in combination with ibrutinib, daratumumab induces direct apoptosis and enhances ibrutinib activity [155] thus, a daratumumab-ibrutinib clinical trial was very recently started (NCT03447808) to evaluate this treatment regimen in patients with symptomatic CLL. CD40 is a member of the tumor necrosis factor (TNF) receptor superfamily expressed by 90–100% of CLL cells [156]. Lucatumumab, an anti-CD40 antibody, mediates ADCC and is well tolerated. However, minimal single-agent activity was reported [157] (NCT00108108).

Anti-BAFF-R: BAFF is a member of the TNF superfamily that supports normal B-cell development and proliferation signaling [158] and the BAFF-receptor (BAFF-R) is often overexpressed in CLL [159]. The CLL microenvironment supports the survival of CLL B-cells by providing pro-proliferation factors and ligands, including BAFF [160]. BAFF/BAFF-R pathway is often over-activated in CLL regardless of treatment [161]. Interestingly, BAFF-driven activation of CLL prompts overexpression of *miR-155* which enhances the responsiveness of CLL cells to BCR signaling and promotes the proliferation of cancer cells [61]. Thus, ianalumab a humanized antibody targeting BAFF-R, was developed. Ianalumab induces ADCC-mediated depletion of B–cells and blocks the BAFF signaling that drives B-cell differentiation, proliferation and survival [161]. Additionally, this drug induces NK response against the targeted cells, and in vitro experiments showed superior ADCC induction when compared with CD20- and CD52-directed antibodies [161]. Ianalumab tolerability was assessed in relapsed/refractory CLL patients (NCT02137889), and is currently under evaluation for combinatory therapy with ibrutinib (NCT03400176).

Anti-ROR1: ROR1 is a receptor of Wnt5a, widely expressed during embryogenesis, but not in normal adult tissues [162]. Recently, Dr. Kipps groups showed that *ROR1* is highly expressed in most cancer cells, including CLL [163]. In CLL cells, ROR1 acts as a Wnt5a receptor to activate the non-canonical Wnt5 pathway signal that leads to proliferation [164]. Higher expression of *ROR1* in CLL is associated with aggressive disease and poor prognosis [163] and recent studies have shown that, along with *BCL2*, *ROR1* is a target of *miR-15a/miR-16-1*, often deleted in CLL [165]. These observations provided the rationale for developing a monoclonal antibody targeting ROR1. Cirmtuzumab is a recently developed humanized monoclonal antibody that targets the extracellular domain of ROR1, blocking the ROR1-mediated signaling and preventing tumor cell proliferation. In a phase 1 clinical trial on relapsed/refractory CLL (NCT02222688), cirmtuzumab was able to inhibit CLL stemness gene expression signatures. The treatment was well tolerated and effective at inhibiting ROR1 signaling. Moreover, a significant reduction of the lymphocyte count was observed, and some patients showed a reduction of CLL cell infiltration. However, no patient showed complete response, indicating that cirmtuzumab may be more effective when used in combination with other drugs [166]. Thus, a clinical trial to evaluate the safety of cirmtuzumab in combination with ibrutinib was started and proved to be well-tolerated and effective, with few cases of complete response (NCT03088878). Since in vitro experiments showed that cirmtuzumab enhances the venetoclax ADCC activity, a clinical trial to evaluate a combination of cirmtuzumab with venetoclax to treat CLL patients is warranted [32]. Both venetoclax and cirmtuzumab were designed to target the genes that are overexpressed in CLL as a consequence of *miR-15a/miR-16-1* loss, thus, the efficiency of this combination therapy provides an additional remarkable indication that microRNAs studies are essential for the development of more effective therapeutic approaches.

#### 4.2.2. CAR-T Cells

Chimeric antigen receptor T therapy (CAR-T) is a type of treatment in which T-cells are withdrawn from the patient (autologous) or from a healthy donor (allogeneic), genetically engineered to express an artificial T-cell receptor designed to bind to a specific antigen on cancer cells, and then infused in the patient. Once re-injected, the CAR-T cells multiply in the bloodstream, generating a population of T lymphocytes that can recognize target cancer cells without Major Histocompatibility Complex (MHC) restriction, and destroy them through cytotoxic effector mechanisms [167]. Indeed, the chimeric receptors combine both antigen-binding and T-cell activating functions into a single receptor. Thus, CAR-T cells destroy the target cells by increasing both cytotoxicity and secretion of cytokines, interleukins and growth factors.

The first approved CAR-T cell approach was based on CD19 antigen targeting. Since CD19 is a marker of B-cell malignancies, CAR-T treatment against CD19 (CTL109 or CART19) has been tested in CLL, acute lymphoblastic leukemia (ALL) and diffuse large B-cell lymphoma (DLBCL) [168,169]. To determine the optimal dose and safety of autologous CART19, a dose optimization (NCT01747486) and a safety-efficacy-cellular kinetics studies were carried on (NCT01029366) showing complete and durable response in about 50% of patients [169,170,171]. Thus, clinical trials were initiated to evaluate CAR-T cells strategy in combination with targeted therapy for the treatment of relapsed/refractory CLLs, such as a CART19-ibrutinib combination, currently under evaluation (NCT02640209). In addition, a clinical trial is currently evaluating the use of ROR1-specific (CAR) T-cells, to treat patients with advanced ROR1 positive malignancies. In this study, T-cells are engineered to specifically recognize and kill ROR1-expressing cancer cells and infused to the patient after conventional therapy (NCT02706392). Lastly, new approaches using engineered Natural Killer cell (CAR-NK) are under investigation for the treatment of CLL (NCT03056339) [172].

#### 4.2.3. Immune Checkpoint Inhibitors: Anti-PD1/PD-L1, Anti-CTLA-4, and Anti-CD47-SIRPα Monoclonal Antibodies

Anti-PD1/PD-L1: An escaping mechanism adopted by cancer cells to suppress host T-cell responses is mediated by the surface protein programmed death-ligand 1 (PD-L1/CD274). In normal conditions PD-L1, expressed on dendritic cells or macrophages, interacts with its receptor PD-1, expressed on activated T-cells, to halt T-cell response and minimize the possibility of chronic autoimmune inflammation [173]. However, in cancer, the PD-1/PD-L1 pathway represents a resistance mechanism for malignant cells to avoid endogenous immune anti-tumor activity. PD-L1 is over-expressed on tumor cells (or on cells of the TME) and by binding to PD-1 receptors on activated T-cells, inhibit their cytotoxic activity [174]. Monoclonal antibodies have been developed to target PD-L1 such as atezolizumab and durvalumab, and PD-1 such as nivolumab and pembrolizumab [175]. Since the efficiency of the immune checkpoint blockade with monoclonal antibodies in solid cancer treatment is remarkable, clinical trials were started to evaluate their use for the treatment of CLL. Atezolizumab is under evaluation for combination with obinutuzumab and venetoclax in relapsed/refractory CLL, SLL, or patients with Richter’s syndrome (NCT02846623), while durvalumab is being tested in combinations with lenalidomide, rituximab, ibrutinib and bendamustine (NCT02733042). Pembrolizumab is under evaluation for the treatment of relapsed/refractory CLL and Richter’s syndrome after ibrutinib treatment. Pembrolizumab induced response in 44% of the patients with Richter’s syndrome with a satisfactory safety profile. However, single-agent pembrolizumab does not have significant therapeutic activity in relapsed/refractory CLL [176]. Therefore, combination with other drugs such as idelalisib or ibrutinib may be more efficient (NCT02332980), and a preliminary study for umbralisib, ublituximab and pembrolizumab combination is showing promising results (NCT02535286). Nivolumab showed an encouraging synergy with ibrutinib in an *in-vitro* experiment [177]. In addition, the results from a phase 1/2 study to evaluate safety, pharmacokinetics, pharmacodynamics and efficacy of the combination of ibrutinib with nivolumab in hematologic malignancies, indicated that this combinatory treatment was particularly beneficial for patients with Richter’s transformation [178] (NCT02329847). An additional active clinical trial (NCT02420912) is evaluating this combination therapy in patients with relapsed/refractory, high-risk untreated CLL, SLL, and Richter’s syndrome. Notwithstanding the promising results, none of these PD-1/PDL-1 inhibitor drugs have been yet approved for CLL treatment.

Anti-CTLA-4: The cytotoxic T lymphocyte-associated antigen 4 (CTLA-4 or CD152) is expressed on normal circulating T-cells, where it acts as a negative regulator to downregulate immune responses [179]. CTLA-4 deficiencies are associated with autoimmune diseases and CTLA-4 agonists are currently used a potential therapy for autoimmune diseases to reduce immune activity [180]. Conversely, blocking CTLA-4 to enhance the immune response toward cancer cells may provide therapeutic benefits for patients [181]. Interestingly, CTLA-4 is expressed in CLL where its activation is associated to increase of survival and proliferation [182]. Ipilimumab was tested in clinical trials accruing patients with several malignancies, including CLL, and showed durable clinical responses in a relatively low proportion of patients (NCT00060372) [183]. In a recently completed clinical trial, a combination of ipilimumab with the toll-like receptor 9 agonist SD-101 and radiation therapy to treat patients with recurrent low-grade B-cell lymphoma showed a partial response in only one patient out of seven, but later had progressive disease (NCT02254772). Thus, further studies are warranted to evaluate this strategy for the treatment of B cell malignancies.

Anti-CD47: CD47 is a cell surface protein with key roles in several cell processes, including apoptosis and phagocytosis. Physiologically, CD47 functions as a marker of “self” by binding to SIRPα on the surface of circulating macrophages to deliver an inhibitory “don’t eat me” signal [184]. CD47 was found overexpressed in leukemia cells as a mechanism of immune evasion [185]. Thus, blocking CD47 may render the cancer cells vulnerable to phagocytosis. Interestingly, the use of specific monoclonal antibodies to bind the CD47 on the surface of CLL cells was shown to also induce caspase-independent cell death [186]. Thus, blocking CD47 may induce both innate and adaptive immune systems to attack the cancer cell. Clinical trials are ongoing to verify the safety of humanized anti-CD47 antibodies [187,188] and among these, Hu5F9-G4, is currently under investigation for the treatment of relapsed/refractory B-cell Non-Hodgkin’s Lymphoma in combination therapy with rituximab (NCT02953509).

#### 4.2.4. Immunomodulatory Drugs (IMiDs)

Immunomodulatory drugs are molecules that boost the immune system response against cancer. The main IMiDs currently under investigation for CLL treatment are Lenalidomide and BNC105P. Lenalidomide induces a reduction of PD-L1 expression on the surface of CLL cells, activates NK-cell response, and restores the cancer-targeting functions of T cell in CLL patients [189,190,191]. In a clinical trial for patients with symptomatic CLL (NCT00602459), lenalidomide was combined with fludarabine and rituximab. Results showed that a lenalidomide consolidation after chemoimmunotherapy is tolerated and extends progression-free survival and overall survival [192]. Thus, clinical trials are ongoing to test the safety of lenalidomide as maintenance therapy for CLL patients after chemotherapy (NCT00774345), and its use as first-line treatment in combinations with bendamustine and rituximab (NCT01400685). Unfortunately, another trial showed elevated toxicity and was stopped (NCT00738829) [193]. A derived of lenalidomide, CC-122, that promotes the degradation of the transcription factors Aiolos and Ikaros, showed effective anti-proliferative, anti-angiogenic and immunomodulatory activities in B-cell lymphoma [194]. Thus a clinical trial to test its activity in CLL when combined with ibrutinib and obinutuzumab was started (NCT02406742). Lastly, BNC105P causes occlusion of tumor vasculature resulting in hypoxia-driven tumor cell necrosis, and thus is defined as “Vascular Disrupting Agent”. BNC105P is converted to BNC105 in activated endothelial cells where it inhibits tubulin polymerization, resulting in a blockage of mitotic spindle formation, cell cycle arrest, and disruption of the tumor vasculature [195]. Interestingly, ibrutinib inhibits the pro-survival BCR signaling of CLL cells in the stromal niche, resulting in their release to the bloodstream. Since ibrutinib-induced lymphocytosis can linger for more than a year, it is possible that CLL cells do not die once they exit the lymph nodes. Furthermore, if the administration of ibrutinib is stopped, the CLL cells return to the lymph nodes. For these reasons ibrutinib efficiency may be increased if combined with a vascular disrupting agent such as BNC105P that would eradicate the CLL cells from the bloodstream, thus prevent them from returning to the lymph node niche. A clinical trial for the use of BNC105P in combination with ibrutinib for the treatment of CLL is currently recruiting (NCT03454165) patients with relapsed/refractory CLL.

## 5. Role of Non-Coding RNAs in Targeted and Immunotherapeutic Strategies for CLL

Target therapy and immunotherapy are showing promising results for the treatment of CLL and other malignancies. However, relapse is often observed as a consequence of the selection of a drug-resistant clone. In addition, cancer cells can develop drug-escaping mechanisms when exposed to single-agent treatment strategies and become resistant, leading to relapse. This observation suggests that early initiation of combinatory treatment may be more efficient in eradicating the disease and that the development of sensitive and accurate detection systems for swift identification of an emerging resistant clone is essential [181]. Several studies showed that signatures of ncRNAs can be used to monitor the progression of CLL and cancer in general. Specifically, microRNAs showed significant practical applications for drug development [15]. In this section, we will discuss their role in the development of novel drugs.

The best example of the key role of non-coding RNAs in the development of new drugs for cancer treatment is provided by the venetoclax, a powerful, specific and well tolerated drug that compensates for the lack of *miR-15a/miR-16-1* targeting of *BCL2* in CLL. Shortly after, cirmtuzumab was developed to compensate for the lack of *miR-15a/miR-16-1* targeting of *ROR1* in CLL. The mechanism of action of these drugs substantiates the essential role of miRNAs in cancer and endorses the study of the molecular profiling of cancer for the identification of more specific targets and the development of less toxic drugs. Interestingly, these drugs could be used in combination to target different pathways in CLL that are dysregulated as a consequence of the same driver event, the loss of *miR-15a/miR-16-1*, as shown by the synergistic effect observed when treating CLL cells from patients [32]. Based on these evidences, leukemic cells with low *miR-15a/miR-16-1* expression, and therefore overexpressing of *BCL2* and *ROR1*, would be more sensitive to combinatory therapy with venetoclax and cirmtuzumab. Remarkably, antisense oligonucleotides against *BCL2* such as SPC2996, have also been tested in a clinical trial to inhibit *BCL2* in CLL (NCT00285103) [203], and ROR1-CART cells are being evaluated for treatment (NCT02706392), further supporting the key role of *BCL2* and ROR1 dysregulation in this disease, driven by the loss of *miR-15a/miR-16-1*.

Another indication of the pivotal role of microRNAs in the development of novel drugs is provided by the development of the monoclonal antibody ianalumab, an anti-BAFF-L which could exert its anti-cancer activity by reducing the expression of *miR-155*. *MiR-155* overexpression has oncogenic activity in CLL, and its dysregulation can be triggered by microenvironment signals such as BAFF binding to its receptor BAFF-L on the CLL cell surface [61]. Interestingly, recent observations from preclinical models suggest that the transfer of *miR-155* via EVs may also contribute to CLL-mediated myeloid-derived suppressor cells (MDSC) induction. MDSC have a key role in the immune suppressive networks in the TME, and the modulation of the TME is under evaluation as a possible strategy for cancer treatment [75,76]. In addition, an inhibitor of *miR-155*, (MRG-106), was developed for the treatment of blood cancers (www.miRagen.com) and it is currently under investigation for safety and tolerability in CLL patients (NCT02580552). Indeed, the generation of synthetic molecules such as miRNA mimics and anti-miRNA molecules for cancer treatment is a very novel approach, and some of these have entered clinical trials, further supporting the outstanding potential of microRNAs for both diagnostic and treatment purposes. The use of ncRNAs as therapeutic agents, however, requires a very different delivery approach when compared to other types of drugs such as small molecules or antibodies. Indeed, small non-coding RNAs need to be delivered to the target cells by a carrier. Liposomal nanoparticles have been used for this purpose. For instance, a *miR-34a* mimic carried by liposomal nanoparticles (MRX34), was tested in a phase I clinical trial in 2013, in patients with primary liver cancer [210] (NCT01829971). Although promising, this study was halted in 2016 because of multiple adverse events. However, other in vitro experiments showed that *miR-34a* restoration can induce cell-cycle arrest and apoptosis [211]. In addition, *miR-34a* targets *NOTCH1*, *E2F1* and *B-MYB*, which promote CLL cell proliferation [212], regulates the NF-κβ signaling in T-cells [213] and has a key role in the immune system response in cancer [214]. Thus therapeutic strategy involving the administration of *miR-34a* mimics in CLL may warrant further studies. Additionally, *miR-34a* downregulation could be a marker for Richter’s syndrome, indicating that its restoration may be beneficial in the treatment of this particularly aggressive disease. Lastly in silico analysis suggested that *miR-34a* targets PD-L1, indicating a role for this microRNA in the regulation of the TME and a possible application as an immunotherapeutic agent [215,216].

*MiR-181b* is also under investigation as a therapeutic agent. *MiR-181b* targets *BCL2*, *MCL1* and *TCL1* genes in CLL [35,54,55] and thus its potential as a therapeutic agent was tested in a CLL mouse model. In this study, an in vivo transfection reagent (in vivo-jetPEI^®^) was used to deliver the synthetic *miR-181b* mimic in Eµ-*TCL1* transgenic CLL mouse model. A significant reduction in the leukemic progression and increase survival of the study cohort was recorded indicating that *miR-181b* could potentially be used to reduce the expansion of CLL B-cells in patients [217]. In addition, *miR-29* [218] and *miR-125a* [219] inhibitors and miRNA mimics are also under investigation for the treatment of different diseases (www.miRagen.com). However, the regulation of *miR-29* and *miR-125* expression in CLL is extremely complex end thus no study has yet evaluated these microRNA as a therapeutic agent for CLL treatment.

Recent studies have suggested that a potential approach for the delivery of small non-coding RNAs in cancer may be the use of EVs [81]. Indeed, sncRNAs can be transferred between cells via EVs, and have different functions [220]. For instance, in vitro experiments showed that EVs from bone marrow stromal cells can rescue CLL B-cells from apoptosis, enhance their migration and induce drug resistance [221]. Thus, the EV-mediated transfer of ncRNAs between cells is currently under investigation [68]. Interestingly, EVs can be artificially designed to deliver their cargo in specific tissues [222] and can be loaded with ncRNAs and other molecules with therapeutic activity [81]. EVs are an intriguing component of TME signaling, and can amplify oncogenic pathways in cancer cells to promote tumor progression, spread, and therapy resistance. Indeed, EVs may be used not only to target cancer cells directly, but also to affect the TME to hinder nursing-like cells from supporting the growth and spread of the tumor, and to prevent the development of resistance [223,224]. Unfortunately, despite the emerging role of EVs as drug delivery systems, safety aspects need to be overcome before introducing this technology to clinical studies [225]. Nonetheless, the study of EVs and TME led to the identification of other potential targets for therapy. Indeed, while studying the ability of leukemia cells to communicate with endothelial cells, Umezu et al. discovered that *miR-92a-3p* is selectively transferred from donor cells to endothelial cells via EVs [72]. More recently, a study on colon cancer showed that EVs containing *miR-92a-3p* can facilitate the endothelial-mesenchymal transition and the angiogenesis process affecting the TME [226]. As mentioned in Section 2, the same molecule is involved in a mechanism of activation of the *VEGF*-based autocrine pathway in CLL B-cells which leads to disease progression and is associated with poor prognosis in CLL [50,51,227]. These observations indicated that an anti-*VEFG* approach may be therapeutically valid. Thus, in vitro experiment were carried out to evaluate the efficacy of *VEGF* inhibitors such as vatalanib and pazopanib in the treatment of CLL [52] and, shortly after, clinical trials for vatalanib (NCT00511043), and for pazopanib (NCT01361334), were started. Unfortunately, the response to pazopanib was limited, showing short term efficacy and low percentage of partial response. Thus, further trials for this drug were discouraged at least as monotherapy for AML [205]. Vatalanib also showed low response rates and concerns for toxicities with high doses [228]. For these reasons, the use of anti-angiogenetic drugs remains widely overlooked in CLL.

**Figure 1:**
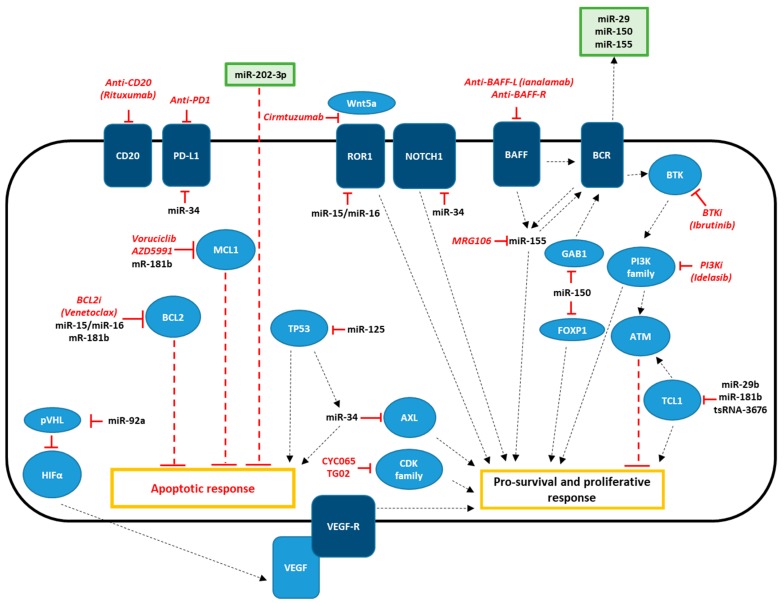
Schematic diagram of effectors, ncRNAs, and drugs involved in CLL therapy. NcRNAs are indicated in black, therapeutic agents are indicated in red. Surface receptors are indicated in dark blue and other effectors are indicated in light blue. Extracellular vesicles are indicated in green.

A simplified scheme of the interactions between microRNAs, their target, and the most common therapeutic agents used in CLL, is reported in Figure 1.

## 6. Conclusions

Altered gene expression in cancer cells is often associated with small non-coding RNA dysregulation. This groundbreaking discovery provided the foundation for the outstanding progress made in the development of novel, more effective drugs for cancer treatment. In CLL, the alteration of miRNA profiles led to the development of Bcl2 inhibitors and anti-ROR1 antibodies, and more targets are currently being studied not only for CLL but also for other malignancies. Altogether, these data indicate that the study of non-coding RNA’s dysregulation in cancer has a pivotal role in the development of novel diagnostic tools and treatment strategies to improve the quality of life of patients.

## Figures and Tables

**Table 1:** Therapeutic approaches for CLL treatment (FDA approved and ongoing clinical trials).

Standard FDA Approved Approaches for CLL Treatment	Type of Therapy/ Clinical Trial Code	Reference
Acalabrutinib	Targeted therapy (BTK) NCT02029443	[94]
BR (bendamustine, rituximab)	Chemo-Immunotherapy NCT02381899	[196]
CG (Chlorambucil, obinutuzumab)	Chemo-Immunotherapy Approved for first-line therapy NCT01010061	[197]
FCR (fludarabine, cyclophosphamide, rituximab)	Chemo-Immunotherapy NCT00090051	[132]
FR (fludarabine, rituximab)	Chemo-Immunotherapy Approved for first-line therapy NCT00860457	[198]
Ibrutinib	Targeted therapy (BTK) Approved for first-line therapy NCT02801578	[91]
Ibrutinib/obinutuzumab	Chemo-Immunotherapy NCT02537613	[199]
Ofatumumab/chlorambucil	Chemo-Immunotherapy NCT00748189	[138]
PCR (pentostatin, cyclophosphamide, and rituximab)	Chemo-Immunotherapy Approved for first-line therapy NCT00049413	[200]
Rituximab/chlorambucil	Chemo-Immunotherapy Approved for first-line therapy NCT00532129	[201]
Rituximab/human hyaluronidase	Chemo-Immunotherapy NCT03467867	[202]
Venetoclax	Targeted therapy (Bcl2) NCT01328626	[118]

**Table 2:** Ongoing clinical trials for novel therapeutic approaches of CLL updated January 2020.

Clinical Trial ID	Treatment	Phase	Status	Date of Start	Reference for Results
NCT00060372	Ipilimumab	I	Completed	04/2003	
NCT00108108	Lucatumumab	I/II	Terminated	04/2005	[157]
NCT00285103	SPC2996	I/II	Completed	06/2005	[203]
NCT00511043	PTK787 (vatalanib)	II	Terminated	11/2005	
NCT00602459	Lenalidomide combined with fludarabine and rituximab	II	Completed	01/2008	[192]
NCT00738829	Lenalidomide combined with fludarabine and rituximab (dose escalation)	I/II	Completed	10/2008	[193]
NCT00774345	Lenalidomide as maintenance therapy for CLL	III	Active	01/2009	[204]
NCT01029366	Autologous CART19	I	Completed	03/2010	[171]
NCT01161511	XmAb5574	I	Completed	10/2010	[142]
NCT01188681	Otlertuzumab in combination with bendamustine	I/II	Completed	10/2010	[151]
NCT01361334	Pazopanib	II	Completed	06/2011	[205]
NCT01400685	Lenalidomide as first line with bendamustine and rituximab	I	Completed	07/2011	
NCT01466153	Inebilizumab in combination with bendamustine or rituximab	II	Completed	02/2012	[206]
NCT01569295	Idelalisib in Combination With Bendamustine and Rituximab	III	Completed	06/2012	[106]
NCT01699152	TG02	I	Completed	09/2012	
NCT01747486	Autologous CART19 (dose optimization)	II	Completed	02/2013	[207]
NCT01829971	miR-RX34 liposomal injection	I	Terminated	04/2013	
NCT02005289	MOR00208 in combination with lenalidomide	II	Active	12/2013	
NCT02137889	Ianalumab	I	Terminated	07/2012	
NCT02222688	Cirmtuzumab	I	Completed	10/2014	
NCT02242942	Obinutuzumab in combination with venetoclax, and obinutuzumab and chlorambucil	III	Active	12/2014	[133]
NCT02254772	Ipilimumab with SD-101 and radiation therapy	I/II	Completed	09/2014	[208]
NCT02329847	Nivolumab with ibrutinib	I/II	Active	03/2015	[178]
NCT02332980	Pembrolizumab in combination with idelalisib or ibrutinib	II	Recruiting	02/2015	[176]
NCT02406742	CC-122 combined with ibrutinib and obinutuzumab	I/II	Active	09/2015	
NCT02420912	Nivolumab and ibrutinib	II	Active	06/2015	
NCT02500407	BTCT4465A (Mosunetuzumab) as a single agent and combined with Atezolizumab	I	Recruiting		
NCT02535286	Ublituximab in combination with umbralisib	I/II	Recruiting	09/2015	
NCT02580552	MRG-106	I	Recruiting	02/2016	
NCT02640209	CART19 with ibrutinib	---	Active	12/2015	
NCT02706392	ROR1-specific CART-cells	I	Recruiting	03/2016	
NTC02733042	Durvalumab in combinations with lenalidomide, rituximab, ibrutinib, and bendamustine	I/II	Active	03/2016	
NCT02742090	Duvelisib	II	Active	04/2016	[109]
NCT02846623	Atezolizumab in combination with obinutuzumab and venetoclax	II	Recruiting	01/2017	
NCT02910583	Ibrutinib plus venetoclax	II	Active	10/2016	
NCT02953509	Hu5F9-G4 in Combination with Rituximab	I/II	Recruiting	11/2016	[209]
NCT02968563	Tirabrutinib and Idelalisib with and Without Obinutuzumab	II	Active	12/2016	
NCT03037645	Vecabrutinib	I/II	Recruiting	04/2017	
NCT03056339	CAR-NK	I/II	Recruiting	06/2017	
NCT03088878	Cirmtuzumab in combination with ibrutinib	I/II	Recruiting	01/2018	
NCT03162536	ARQ-531	I/II	Recruiting	07/2017	
NCT03218683	AZD5991 with or without venetoclax	I	Recruiting	10/2017	[127]
NCT03336333	BGB-3111 (zanubrutinib)with Bendamustine plus Rituximab	III	Recruiting	11/2017	
NCT03400176	Ianalumab with ibrutinib	I	Recruiting	04/2018	
NCT03447808	Daratumumab with ibrutinib	I	Recruiting	07/2018	
NCT03454165	BNC105P in combination with ibrutinib	I	Recruiting	03/2018	
NCT03572634	TP-0903	I/II	Recruiting	06/2019	
NCT03734016	Zanubrutinib (BGB-3111) versus Ibrutinib	III	Active	11/2018	
NCT03739554	CYC065 and venetoclax	I	Recruiting	01/2019	
NCT03740529	LOXO-305	I/II	Recruiting	11/2018	
NCT03823365	Blinatumomab	I	Recruiting	12/2018	
NCT03824483	Zanubrutinib, obinutuzumab, and venetoclax	II	Recruiting	02/2019	
NCT04116437	Zanubrutinib (BGB-3111)	II	Recruiting	10/2019	
